# Accelerating Evaluation of Financial Incentives for Fruits and Vegetables: A Case for Shared Measures

**DOI:** 10.3390/ijerph182212140

**Published:** 2021-11-19

**Authors:** Nadine Budd Nugent, Carmen Byker Shanks, Hilary K. Seligman, Hollyanne Fricke, Courtney A. Parks, Sarah Stotz, Amy L. Yaroch

**Affiliations:** 1 Gretchen Swanson Center for Nutrition, Omaha, NE 68114, USA; cbshanks@centerfornutrition.org (C.B.S.); hfricke@centerfornutrition.org (H.F.); cparks@centerfornutrition.org (C.A.P.); ayaroch@centerfornutrition.org (A.L.Y.); 2 Division of General Internal Medicine, University of California, San Francisco, CA 94143, USA; hilary.seligman@ucsf.edu; 3 Centers for American Indian and Alaska Native Health, University of Colorado Anschutz Medical Campus, Colorado School of Public Health, Aurora, CO 80045, USA; sarah.stotz@cuanschutz.edu

**Keywords:** financial incentives, fruits and vegetables, nutrition incentives, produce prescriptions, food security, food access, food assistance, GusNIP, evaluation

## Abstract

Food insecurity, or lack of consistent access to enough food, is associated with low intakes of fruits and vegetables (FVs) and higher risk of chronic diseases and disproportionately affects populations with low income. Financial incentives for FVs are supported by the 2018 Farm Bill and United States (U.S.) Department of Agriculture’s Gus Schumacher Nutrition Incentive Program (GusNIP) and aim to increase dietary quality and food security among households participating in the Supplemental Nutrition Assistance Program (SNAP) and with low income. Currently, there is no shared evaluation model for the hundreds of financial incentive projects across the U.S. Despite the fact that a majority of these projects are federally funded and united as a cohort of grantees through GusNIP, it is unclear which models and attributes have the greatest public health impact. We explore the evaluation of financial incentives in the U.S. to demonstrate the need for shared measurement in the future. We describe the process of the GusNIP NTAE, a federally supported initiative, to identify and develop shared measurement to be able to determine the potential impact of financial incentives in the U.S. This commentary discusses the rationale, considerations, and next steps for establishing shared evaluation measures for financial incentives for FVs, to accelerate our understanding of impact, and support evidence-based policymaking.

## 1. Introduction

### 1.1. Background

In 2019, one in ten United States (U.S.) households were food insecure [1]. The COVID-19 pandemic led to job losses and financial strain for millions of Americans, resulting in massive increases in food insecurity, of which current estimates surpass one-third of all households [2,3]. Food insecurity and its resulting constraints on food purchases, including fruits and vegetables (FVs), is disproportionately experienced by populations with low income and/or who are ethnic minorities. Through multiple complex mechanisms, food insecurity has been linked with higher risk of obesity, cardiovascular disease, type 2 diabetes, hypertension, asthma, depression, and mental illness [4,5]. Additionally, individuals who live in food-insecure households have higher healthcare expenditures (~$1800 yearly) than those in food-secure households [6]. Adequate daily FV intake (FVI), 1.5–2 cups and 2–3 cups, respectively, is essential to prevent chronic disease morbidity and mortality. Currently, only one in ten Americans meet U.S. guidelines for FVI [7]. Annual healthcare spending attributed to suboptimal diets exceeds $50 billion dollars per year [8]. These data suggest a public health crisis that is driving disparities in diet-related chronic disease, which warrants federal action and systemic solutions.

### 1.2. Financial Incentives as a Policy-Driven Solution

The Supplemental Nutrition Assistance Program (SNAP) is the largest domestic federal food assistance program, serving 42 million (1 in 7) low-income Americans annually. It is well established that food prices are one of the most important factors determining individual food choices [9]. A recent USDA-sponsored, nationally representative survey found that 61% of SNAP participants reported that food costs were a barrier to achieving a healthy diet [10]. For the first time since 2006, the Thrifty Food Plan, which serves as the basis for establishing maximum SNAP benefit allotments, was reevaluated this year and increased maximum benefits by $4.79 per day for a family of four to more realistically represent current costs of nutrient-dense foods and beverages that can be purchased on a limited budget to meet national dietary guidelines [11]. In addition, many experts have called for structural changes to the program to better address poor dietary intake among beneficiaries [12]. One suggested modification to SNAP has been the implementation of financial incentives to encourage the purchase of more healthful foods, including FVs. Financial incentives also stimulate local economies, making them an attractive solution in the context of U.S.’s recovery from the COVID-19 pandemic.

Financial incentives for FV purchases have previously been funded through competitive grant awards through the U.S. Department of Agriculture (USDA), including the Healthy Incentives Pilot (HIP) and the Food Insecurity Nutrition Incentive (FINI) program [13,14]. The newest USDA iteration, the Gus Schumacher Nutrition Incentive Program (GusNIP), began in 2019 and was created through legislation in the 2018 Farm Bill. Other federally supported financial incentive programs include the WIC Farmers’ Market Nutrition Program (WIC FMNP) [15], which provides coupons to participants of the Special Supplemental Nutrition Program for Women, Infants, and Children (WIC) to purchase fresh, locally-grown FV at farmers markets or similar settings, and the Senior FMNP [16], which provides similar supports for seniors (aged 60 or older) experiencing low income. However, benefits are limited to $30 and $50 per recipient per year for WIC FMNP and senior FMNP, respectively. Other financial incentive funders outside of USDA include health-focused foundations, healthcare systems, and local/state government agencies.

GusNIP supports nutrition incentive (NI) and produce prescription (PPR) projects. Within these project types, grantees are funded to implement pilot, mid-sized, or large-scale NI or PPR projects. Notably, there has been enormous variation in the implementation of GusNIP. Some variables that differ include project model type (PPR, NI), scope, setting, demographic context, populations served, incentive delivery (e.g., token, automatic discount), eligible FVs (e.g., fresh, canned, frozen), accompanying nutrition education, and evaluation methodology.

NI projects seek to increase the purchase and consumption of FV among SNAP participants. Typically, SNAP participants receive incentives (e.g., vouchers, coupons, automatic discounts) to purchase FV for each SNAP dollar spent at the point of purchase when shopping at participating retailers (e.g., farmers markets, grocery stores). By providing direct cash to local businesses and farmers, NI projects are hypothesized to boost employment, and increase local spending, much like an economic stimulus.

PPR projects seek to increase FV purchases and FVI among patients in coordination with their healthcare provider. Patients must meet certain eligibility criteria based on household income, household food-security status, and/or an existing chronic health condition(s) or risk factor(s). In PPR projects, healthcare providers “prescribe” FVs to patients, who can exchange or “redeem” the prescription (e.g., voucher) for produce at participating food retailers or in-house “food pharmacies.” Although PPR projects also provide direct cash flow into the local economy, they traditionally have fewer participants (i.e., lower reach) but more substantial benefits (i.e., higher dose). They tend to be implemented within multi-component interventions that include nutrition education and frequent contact with the healthcare system.

Since 2019, GusNIP has funded 52 unique projects in 26 states and DC. Simultaneously, a National Training, Technical Assistance, Evaluation, and Information Center (NTAE) was added to support GusNIP grantees with implementation, outreach and communications, and reporting and evaluation. In April 2021, Congress announced an additional $75 million to support current FINI and GusNIP awardees in providing pandemic relief through the GusNIP COVID Relief and Response grants program (GusCRR). To date, 35 additional projects or project expansions have been supported by GusCRR.

### 1.3. Evidence about the Impact of Financial Incentives for FVs

Financial incentives for FVs are hypothesized to positively impact individual dietary intake and health, healthcare costs, and local economic growth [17,18,19]. To date, most research has focused on evaluation of individual programs, utilizing a wide array of quasi-experimental (e.g., cross-sectional, pre-post) and experimental (e.g., randomized controlled trial) study designs and ranging from single sites (e.g., farmers market) to multiple states or territories (e.g., Navajo Nation). These studies have shown increases in FV purchases and FVI and improvements in psychosocial measures, food security, and clinical markers, such as BMI and blood sugar levels [20,21,22]. Economic models suggest that financial incentives could reduce chronic disease morbidity, mortality, and healthcare costs over the long term [23]. For example, a 2019 microsimulation study estimated that financial incentives could prevent 1.9 million cardiovascular events, reclaim 4.6 million quality-adjusted life-years (QALYs), and save $39.7 billion in healthcare costs [24]. Other models have also shown societal cost savings [23,25] and increased employment, labor income, and economic development [26].

A 2020 scoping review found 19 evaluations of NI projects serving SNAP participants [18]. All but one study demonstrated positive associations with either FV purchases or FVI. However, the authors stated that the rigor of these evaluations was poor, and variations in program structure make it difficult to understand which program elements were most effective to improve food behaviors and overall health [18]. Similarly, a 2019 scoping review of NI projects showed that price incentives resulted in modest improvement in dietary outcomes and food-related behaviors, but variability in measurement and definition of outcomes made it difficult to summarize the available evidence across projects [19]. Most studies were limited by small sample sizes and narrow geographic areas, making generalizability low [19]. A 2019 systematic review on food-insecurity interventions in healthcare settings, including PPR, found no effect on FVI when individual studies were pooled, while overall impact on health or healthcare utilization could not be determined due to variability in measures [17]. Finally, a recent meta-analysis on PPR found a 22% increase in FVI among participants when estimates were pooled; however, the authors cautioned that considerable heterogeneity and other methodological limitations preclude causal inference [27].

Evaluations of federally funded financial incentive projects have yielded similarly ambiguous conclusions. From 2011–2012, HIP was implemented in Hampden County, MA, and evaluators found that SNAP participants receiving a 30% incentive towards the purchases of FVs consumed 0.24 cups more FVs per day compared to SNAP participants who did not receive the incentive [13]. Evaluation of the subsequent FINI program (2015–2017) found no detectible change in FVI among participants [28]. However, another evaluation of a subset of FINI programs, representing 76 farmers markets across 13 states and DC, reported that nutrition incentives issued to SNAP shoppers at farmers markets had statistically significant, positive effects on FVI for those given the highest incentive level (spend $1 with SNAP, receive $2 for FV) by 0.16 cups/day [29].

Heterogeneity of project types and evaluation methodology has made it difficult to determine which incentive models are most effective and under what conditions. For example, no concrete evidence exists about what dose and frequency is needed to result in behavior change; for instance, what incentive amounts, delivery mechanisms, program types, and supplementary approaches (e.g., education, transportation services) are most effective and in which settings and populations. Shared measures across financial incentive projects would accelerate understanding of impact and support evidence-based programming and policies. A 2018 qualitative evaluation of FINI reported that grantees (*n* = 19) cited the lack of scientifically robust and coordinated evaluations of financial incentives as a challenge in understanding best practices in varying contexts [30]. Grantees also expressed strong interest in advancing consistent measurement across projects but cited lacking capacity and coordination to do so [30,31].

## 2. Aims and Approach

In this paper, we provide current evaluation strategies for financial incentive programs and the rationale, considerations, and next steps for establishing shared evaluation measures these programs and particularly for GusNIP projects. The NTAE is situated to provide infrastructure and support necessary to create a national evaluation model. We describe the process of the GusNIP NTAE, as a federally-supported initiative, to identify and develop shared measures to be able to determine the potential impact of financial incentives in the U.S. This includes development of common goals across partners, ongoing communication, communities of practice that allow for peer learning and support, and technical assistance. While impact on youth, systems, and society at large are important evaluation components, they are beyond the scope of this paper. Additionally, WIC and senior FMNP are not the focus of this article, but it is important to note neither FMNP program utilizes standardized measures to evaluate impact and is a limitation that has been cited in the peer-reviewed literature [32]. Herein, we focus on individual- or organizational-level outcomes for adults participating in GusNIP PPR or NI projects.

## 3. Discussion

### 3.1. Making the Case for Shared Measures for Financial Incentive Projects

A recent AJPH commentary called for an expansion of NI programs to include all SNAP participants in the U.S., while highlighting insufficient federal funding [33]. Yet, an impediment to expansion is the lack of clarity around which models have the greatest public health impact and at what cost. Financial incentive projects can be led by different types of organizations (e.g., non-profits, food banks, healthcare systems, governments, academic institutions), which have varying levels of experience and capacity for evaluation. Many projects do not have adequate expertise or capacity to design sampling frames; create data collection instruments, codebooks, and protocols; conduct analyses; and interpret, contextualize, and disseminate findings. Evaluation is hampered because associated costs are commonly drawn from funds that could be allocated to participants (i.e., additional incentives) or for project delivery. Project implementers may also have to satisfy evaluation requirements from multiple funders, each of which may have different required measures and outcomes of interest.

The result of these challenges is a lack of data about the national impact of financial incentive projects, even though many are federally funded under a single mechanism. Shared measures would allow for the comprehensive evaluation of federal incentive projects by aggregating data while allowing for meaningful comparisons of main outcomes (e.g., individual FVI, household food insecurity) across and within projects. Shared measures would support elucidation of best practices, such as the most effective incentive dose or co-intervention (e.g., transportation support) for a variety of geographical settings and populations served. Standardized survey modules and other instruments help to eliminate “guesswork” and build capacity for practitioners that are less familiar with evaluation [30,33]. Shared and standardized evaluation measures allow for multiple stakeholders to understand how projects compare and can provide evidence for funders who are interested in capturing return on investment.

### 3.2. Considerations for the Selection of GusNIP Shared Measures

Evaluation of GusNIP financial incentive projects requires a hybrid approach that balances capacity and rigor. Since the evidence base is being established in “real-time” as projects are implemented, such as in natural experiments, evaluations often cannot follow a more formal, rigorous path (e.g., employing RCTs). The aim of academic research is to generate new, generalizable knowledge in the field, and selection of validated instruments appropriate for the target population is often prioritized, despite added costs and resources to administer these instruments. On the other hand, program evaluation generally focuses on the goal of continually improving a particular project. As such, process measures (e.g., customer satisfaction, number of participants over time (reach), number of education sessions delivered (dose)) are generally prioritized, both because the goals of evaluation are different and because programmatic evaluations are often constrained by time, money, and personnel. Thus, selecting shared measures requires a balance of multiple factors, including:

- Adaptability to populations with limited literacy, lack of English proficiency, and diverse foodways;
- Ability to translate into languages other than English;
- Ability to contextualize findings with secondary data sources often used in surveillance efforts (e.g., NHANES, BRFSS);
- Capitalizing on commonly used measures among previous and current grantees to reduce burden;
- Alignment with USDA’s required metrics; and
- Ease of administration relative to grantee evaluation capacity.

### 3.3. Which Outcomes Need Shared Measures?

**Figure 1:**
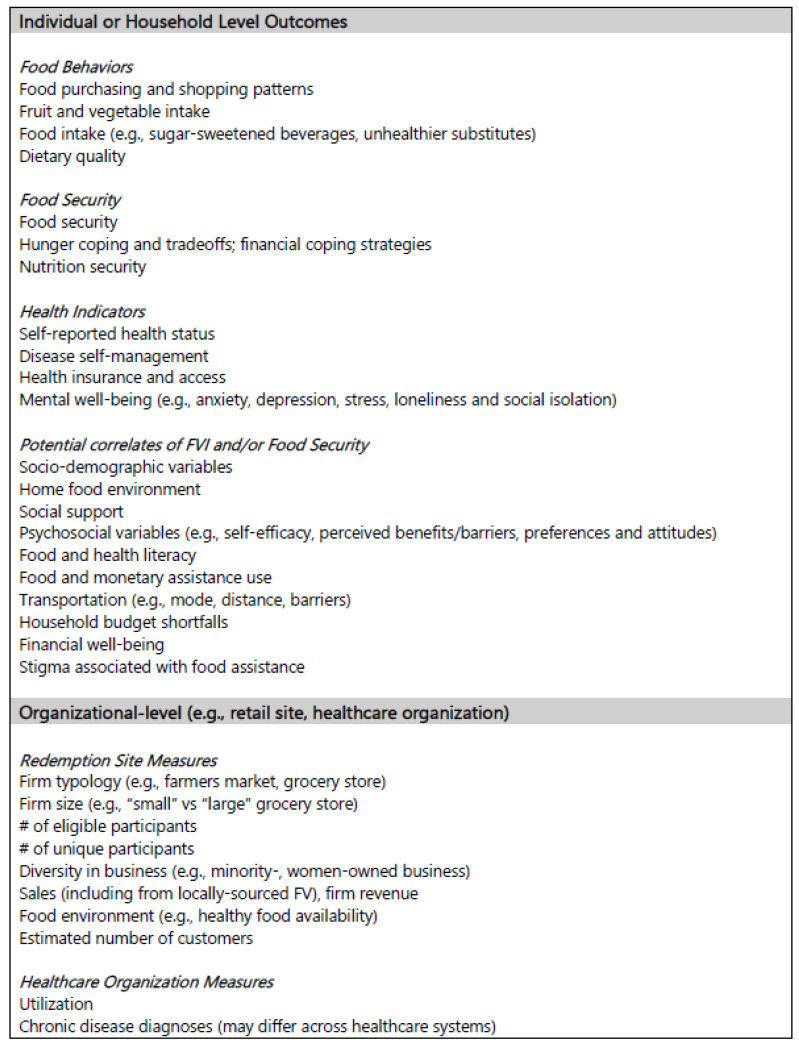
Outcomes needing consensus on appropriate shared measures for financial incentive programs. There are several other important outcomes than those listed here, however, those listed could benefit from a consensus on an appropriate shared measure.

To evaluate the impact of financial incentive projects, several objective measures can be applied. For example, common measures include transactional data to assess food purchasing trends, clinical markers to identify changes in health status, and clinical diagnosis from an electronic health record (EHR) in conjunction with International Classification of Diseases codes to assess associated healthcare costs from claims data of a particular diagnosis. Pooling of these objective measures requires some level of “shared best practices” for data collection, coding, and interpretation. Where such measures are not available, alternative measures may become essential. For example, using food-purchasing data has inherent strengths due to its objectivity and automation in SNAP-approved settings through Electronic Benefit Transfer (EBT) cards. However, in smaller retail outlets (e.g., corner stores), receipt data collection may not be possible, so more subjective measures (e.g., vendor or store procurement) may be an appropriate alternative. Essential shared measures at the individual level include dietary intake, household food insecurity, food shopping patterns, program utilization and satisfaction, home food environment, psychosocial variables, and other intermediate variables (Figure 1). Sociodemographic measures should be updated to be consistent with recommendations for gender and LGTBQIA+ inclusivity, and FV screeners using algorithms to calculate quantities (e.g., FV cup equivalents, servings) should account for non-binary/third gender when sex-age coefficient inputs are required.

Organizational-level data from program implementers, redemption sites, and healthcare organizations can provide meaningful process-evaluation data on participant eligibility and enrollment, incentive issuance and redemption, overall sales of targeted foods and their replacements (to understand potential substitution effects), and contextual factors that influence incentive redemption and FV purchases, such as incentive dose (e.g., amount and frequency) and supplementary services offered. Most organizational-level measures are objective and relatively easy to collect; however, some have associated difficulties. For example, typologies on redemption site type (e.g., “grocery store”, “small food store”) differ among subject matter experts and practitioners and may affect interpretations of the best locations to implement NI/PPR projects. Other organizational-level measures have proven difficult to track (e.g., number of eligible participants), and in these circumstances, consensus on appropriate proxy measures should be reached. Financial impact of GusNIP on program operators, including vendors, farmers, store-, and other business owners, is a current gap in the field, but objective data (e.g., revenue) are exceedingly hard to collect, as many retailers consider such data as proprietary. Thus, considerations for a valid, standardized proxy measure should be developed. Last, streamlined reporting systems should be established and adopted by grantees and partner organizations (e.g., vendors, retailers) so that redemption metrics and other firm-level data are reported consistently and uniformly across projects.

Standardized measures for health outcomes and healthcare utilization are challenging to establish due to complexity in retrieving claims and EHR data, variations across EHR systems, and differences in outcomes of interest among different population groups (e.g., hemoglobin A1c among patients with diabetes vs. BMI among patients with obesity). For PPR projects, claims data can sometimes establish a comprehensive view of a patient’s interactions with the healthcare system, identify costs or diagnoses attributed to a healthcare visit, and provide information on medication adherence. However, such data are owned by insurance companies or payers (e.g., Medicaid or Medicare), are difficult to retrieve due to privacy laws, only reflect data attributed to a fee (the “claim”), and are temporally limited, capturing only a snapshot in time versus a person’s entire health history. EHR data, on the other hand, can provide rich contextual data (e.g., anthropometric data, biochemical indices, behavioral outcomes, notes) and track patient progress over time but can only capture a patient’s activities within that clinic or health system. EHR and claims data are most useful when used in tandem to obtain a complete picture of a participant’s health status [34]. It is important to better understand how to interact with these two types of data within the context of evaluating financial incentive projects; reach consensus on how to define common outcomes, such as “utilization” (e.g., emergency department visits, 30-day readmissions, appointment no-shows); and standardize diagnoses definitions across healthcare organizations.

## 4. Conclusions

The development of shared measures to evaluate financial incentive projects, specifically federally funded projects, such as GusNIP, will help justify future funding and support evidence-based programming and policy. A robust shared national evaluation will allow for identifying common elements across different settings to assess process and impact and inform iterative program improvements. The NTAE is well-positioned to provide a singular open source of guidance and resources on program evaluation for grantees and other practitioners. For organizational-level reporting, technology solutions are currently under development to provide a password-protected portal for grantees and partner organizations to report their data securely and easily while reducing redundancy and allowing insight into past trends and performance through data visualization. A timeline of NTAE activities involved in developing shared measures and reporting systems is shown in Table 1 One of the first steps in this process is the development of a compendium of shared measures for key constructs associated with the impact of GusNIP financial incentive projects, including the rationale for why each measure was selected, if and how each measure can be adapted for specific audiences and contexts, and guidance for administration. The NTAE aims to reach consensus on the most appropriate shared measures for each construct of interest through a collaborative approach. This approach includes collecting input from an advisory committee, researchers, and practitioners; consideration of current practices in research and evaluation; a review of the latest peer-reviewed research; adherence to current GusNIP RFA requirements; and consideration of capacity among grantees to conduct evaluation. Since its inception, the NTAE has incorporated participatory research principles in the development and implementation of shared measures and related resources. Specific examples of participation with grantees and other partners include feedback on initial data collection drafts via surveys, open-forum webinars, and 1:1 conversations with NTAE staff; creation of an evaluation subcommittee and an external evaluators’ community of practice to share concerns, elevate successes, and improve existing resources; and peer-reviewed case studies featuring grantee programs that address key nuances, barriers, and facilitators to financial incentive projects, among other activities. The NTAE intends to continue exercising participatory approaches with an emphasis on iterative feedback from all key parties involved.

As the evidence base grows, details on the most effective program attributes and complementary approaches will emerge. This evidence will build the foundation for future direct comparisons using more rigorous study designs with larger sample sizes among more diverse populations to improve external validity of the findings. Lastly, systematic reviews and meta-analytic approaches can be used to summarize findings and make comparative and pooled inferences of impact. By developing, defining, and housing shared measures on a public website accompanied by guidance on how to use them, practitioners and researchers alike will have access to requisite resources regardless of whether they are funded by GusNIP. In this way, the NTAE may contribute to efforts to accelerate research on the impact of financial incentives for FVs more broadly and support continued efforts to increase access to FV for households experiencing low income and food insecurity.

## Figures and Tables

**Table 1:** Timeline of key NTAE-led activities for establishing shared measures and reporting systems for GusNIP programs.

Activity	Time Period	Collaborators	Description
GusNIP Award Announcements	- September 2019	N/A	- Inaugural GusNIP projects officially begin - GusNIP NI and PPR projects range from 1–4 years - GusNIP NTAE Center is a 4-year collaborative agreement award
**Shared Measures Development (participant and organizational)**			
Literature Reviews of Financial Incentive Programs	- September 2019-ongoing	NTAE, External Evaluation Experts	- Begin literature review on current evaluation measures used to evaluate produce prescription and nutrition (i.e., SNAP) incentive programs to inform core measure selection - Review participant survey modules used in nationally represented surveys (e.g., NHANES, BRFSS) to assess food behaviors - Draft a PPR systematic literature review on current evaluations, gaps, and areas of opportunity of produce prescription programs - Draft a NI systematic literature review on current evaluations, gaps, and areas of opportunity of nutrition incentive programs - Develop the Resource Library, a searchable database of grey literature and practitioner resources (e.g., reports, infographics, toolkits, briefs)
Evaluation Partner Calls	- September 2019-ongoing	NTAE, External Evaluation Experts, USDA NIFA	- Engage with external evaluation experts that provide scientific, statistical, and reporting support to NTAE reporting & evaluation team. - Conduct twice monthly calls to plan and develop evaluation-related resources and/or analyses with external evaluation experts
Grantee Narrative Reviews	- October–November 2019	NTAE, External Evaluation Experts, 2019 GusNIP Grantees	- Collate grantee narratives to compare and aid selection of proposed measures to establish process and impact evaluation
Initial Grantee Calls	- November–December 2019	NTAE, External Evaluation Experts, 2019 GusNIP Grantees	- Assess capacity and plans for evaluation, including measures used and study design, to inform core measure selection for participant surveys
NTAE and Nutrition Incentive Hub Orientation & Kickoff	- 15–17 December 2019	NTAE, Nutrition Incentive Hub Partners *	- Establish mission, goals, and activities of the Nutrition Incentive Hub - Solidify outcomes needing shared measures - Begin core measure selection contingent on input from an advisory committee, researchers, and practitioners (i.e., ‘External Evaluation Experts’); consideration of current practices in research and evaluation; a review of the latest peer-reviewed research; adherence to current GusNIP RFA requirements; and consideration of capacity among grantees to conduct evaluation
Grantee Site Visits	- January–March 2020	NTAE, Nutrition Incentive Hub Partners, External Evaluation Experts, 2019 GusNIP Grantees	- Conduct site visits with a sample of Grantees to inform evaluation approaches (new visits postponed since March 2020)
Conduct Sample Size Calculations and Sampling Strategy	- February 2020	NTAE, External Evaluation Experts	- Develop sampling strategy across Grantees to ensure adequate sample sizes and power for GusNIP NI and PPR projects
**Reporting Systems for Organizational-level Metrics**			
Develop and Implement Interim Grantee Reporting Systems	- September 2019-August 2021	NTAE, Contracted Digital and Technology Consultants, GusNIP Grantees	- Develop versatile, interim solution to systematically collect core metrics from grantees and firms using Smartsheet, a secure, cost-effective, cloud-based platform and consumer-level reporting using Qualtrics - Develop profiles and dashboards for each grantee showing reporting status, program distinctions, and data highlights - Create trainings and provide 1:1 support - Develop a streamlined system to export, securely store, and clean data prior to analysis - Develop data migration protocols to transfer data/content from Smartsheet to secure portal - Tailor Qualtrics participant surveys for grantees
Develop and Launch Interim Website for Grantees and Other Practitioners	- September 2019–March 2020 - Interim Website launch March 2020	NTAE, Nutrition Incentive Hub Partners, Contracted Digital and Technology Consultants	- Develop a public website for grantees, practitioners, and collaborators - Share resources and core metrics for GusNIP grantees and projects
Website Development and Launch	- Development October 2020 to August 2021 - Website launch August 2021	NTAE, Nutrition Incentive Hub Partners, Contracted Digital and Technology Consultants	- Complete content migration (interim to new website) - Launch a new public website with resources for grantees and practitioners and information about the NTAE and the collaborative network of organizations involved in the Nutrition Incentive Hub
Secure Web Portal Development and Launch	- Development October 2020 to August 2021 - Secure Portal launched on August 2021	NTAE, Nutrition Incentive Hub Partners, Contracted Digital and Technology Consultants, GusNIP Grantees	- Finalize scope of work for development of new secure portal - Migrate more than 20,000 grantee firm-level reports from an interim data collection system to the secure portal - Provide training and support resources to grantees, partners, firms, and technical assistance providers - Incorporate data visualization and enhanced data analysis of GusNIP-wide aggregate data through Power BI through grantee- and firm-level dashboards - 1235 user accounts that have been registered, representing 59 GusNIP grantees and 2653 firms
**GusNIP Grantee Training and Peer-Learning Opportunities**			
Grantee Introductory & Training Webinars	- September 2019-ongoing - Occurs annually with all incoming grantees	NTAE, Nutrition Incentive Hub Partners, External Evaluation Experts, GusNIP Grantees, USDA NIFA	- Introduce the Nutrition Incentive Hub and services provided - Disseminate core minimum datasets for NI and PPR projects, including standardized shared firm and participant-level survey metrics
Grantee Onboarding	- September 2019-ongoing	NTAE, Nutrition Incentive Hub Partners, External Evaluation Experts, GusNIP Grantees	- Develop and implement a Program Advisor (PA) model, whereby each grantee is matched with a NTAE reporting & evaluation team member who serves as their main contact during the grant period - Provide tailored services and training opportunities for each grantee to implement and evaluate their programs (including organizational- and participant-level metrics)
Grantee Resource Development for Evaluation	- September 2019-ongoing	NTAE, Nutrition Incentive Hub Partners, External Evaluation Experts, GusNIP Grantees and Partners	- Develop print and video resources to assist with project evaluations, based on grantee needs and requests - Expand evaluation resources for PPR projects such as data sharing agreements, health measures protocols, and engaging with external physician scientist consultants (i.e., external evaluation experts)
GusNIP Nutrition Incentive Hub Annual Convening	- 2019–2023 - Occurs annually	NTAE, Nutrition Incentive Hub Partners, External Evaluation Experts, USDA/NIFA, GusNIP Grantees and Partners	- Host annual 3-day intensive practitioner convening, with GusNIP grantees and other practitioners in sessions around reporting and evaluation, engagement with USDA NIFA staff, incentive technology, and COVID-19 response strategies - The 2021 Convening was virtual and included 979 participants across 5 tracks, 40 sessions, and featured 125 speakers
GusNIP Discussion Groups	- April 2020-Ongoing	NTAE, Nutrition Incentive Hub Partners, External Evaluation Experts, GusNIP Grantees and Partners	- Peer-to-peer learning for GusNIP NI and PPR practitioners on Slack and GusNIP portal
External Evaluator Community of Practice	- July 2021-Ongoing	NTAE, Nutrition Incentive Hub Partners, External Evaluation Experts, GusNIP Grantees and Partners	- Foster bi-directional communication between grantees and their evaluators and the GusNIP reporting & evaluation team - Share best practices to inform organizational- and participant-level measurement - Generate ideas for collaborative evaluation practices, such as peer-reviewed manuscripts and resource development
Evaluation Working Group	- September 2021-Ongoing - Occurs every other month	NTAE, Nutrition Incentive Hub Partners, External Evaluation Experts	- Develop best practices and evaluation tools for a variety of NI and PPR collaborators (e.g., store owners, market managers, healthcare providers) to conduct evaluation and disseminate meaningful results - Pilot evaluation tools with grantees for ease of use/feasibility - Coordinate the development of resources on evaluation, informed by grantee requests and gaps in the broader NI and PPR fields
Evaluation Funding Support	- 2019–2023 - Occurs annually	USDA NIFA, NTAE, Nutrition Incentive Hub Partners, GusNIP Grantees and Partners	- Award “Capacity Building and Innovation Mini-grants” to GusNIP grantees (can be used to assist and enhance evaluation) - Provide funding support to Grantee applicants for data collection needs (e.g., participant survey stipends, tablets for data collection)
**Building the Evidence for Financial Incentive Programs**			
GusNIP Case studies	- September 2019-ongoing	NTAE, Nutrition Incentive Hub Partners, External Evaluation Experts, GusNIP Grantees and Partners	- Develop and implement case studies on various aspects of evaluation, in collaboration with current GusNIP grantees and their partners
Grantee In-depth Interviews	- March–May 2020	NTAE, External Evaluation Experts, GusNIP Grantees and Partners	- Collect, analyze, and disseminate results on the impact of COVID-19 on 2019 GusNIP grantees’ project implementation & evaluation
Interim GusNIP Impact Analyses	- August 2020-ongoing	NTAE, External Evaluation Experts, GusNIP Grantees and Partners, USDA NIFA	- Clean, aggregate, and analyze yearly organizational-level data to determine interim impact of GusNIP NI and PPR projects - Clean, aggregate, and analyze yearly participant-level data to determine interim impact of GusNIP NI and PPR projects - Develop and disseminate individualized grantee impact reports - Develop and disseminate peer-reviewed publications on impact of GusNIP NI and PPR projects - Present results at nationally represented conferences - Prepare and submit annual report to Congress
PPR Project Impact Sub-studies	- 2022–2023	NTAE, External Evaluation Experts, GusNIP Grantees and Partners	- Develop and implement rigorous sub-studies to determine participant-level impact of GusNIP PPR projects - PPR sub-studies will include a sub-sample of GusNIP grantees and employ robust study designs (e.g., cohort, with control group)
NI Project Impact Sub-studies	- 2022–2023	NTAE, External Evaluation Experts, GusNIP Grantees and Partners	- Develop and implement rigorous sub-studies to determine participant-level impact of GusNIP NI projects - NI sub-studies will include a sub-sample of GusNIP grantees and employ robust study designs (e.g., cohort, with control group)
Conduct Comprehensive GusNIP Impact Evaluation	- 2019–2023	NTAE, External Evaluation Experts, GusNIP Grantees and Partners, USDA NIFA	- Clean, aggregate, and analyze multi-year, multi-project organizational- and participant-level data to determine comprehensive impact of GusNIP NI and PPR projects - Develop and disseminate peer-reviewed publications on impact of GusNIP on grant program objectives (e.g., FV consumption, food security) for NI and PPR projects - Present results at nationally represented conferences - Prepare and submit final report to Congress
2019–2023 NTAE close-out	- 31 August 2023		- Inaugural GusNIP NTAE (2109–2023) concludes - Announcement of 2023–2026 NTAE awardee

* The Nutrition Incentive Hub, created by the GusNIP Training, Technical Assistance, Evaluation, and Information Center (NTAE), is a coalition of partners to support NI and PPR projects. For more about partners, please visit: https://www.nutritionincentivehub.org/about/partners/overview, accessed on 15 November 2021.
